# Complex Electromagnetic Fields Reduce *Candida albicans* Planktonic Growth and Its Adhesion to Titanium Surfaces

**DOI:** 10.3390/biomedicines9091261

**Published:** 2021-09-18

**Authors:** Simonetta D’Ercole, Silvia Di Lodovico, Giovanna Iezzi, Tania Vanessa Pierfelice, Emira D’Amico, Alessandro Cipollina, Adriano Piattelli, Luigina Cellini, Morena Petrini

**Affiliations:** 1 Department of Medical, Oral and Biotechnological Sciences, University “G. d’Annunzio” Chieti-Pescara, Via dei Vestini 31, 66013 Chieti, Italy; gio.iezzi@unich.it (G.I.); tania.pierfelice@unich.it (T.V.P.); emira.damico@unich.it (E.D.); apiattelli@unich.it (A.P.); morena.petrini@unich.it (M.P.); 2 Department of Pharmacy, University “G. d’Annunzio” Chieti-Pescara, 66013 Chieti, Italy; silviadilodovico@unich.it (S.D.L.); l.cellini@unich.it (L.C.); 3 Independent Researcher, 92019 Sciacca, Italy; alexandros1960@libero.it; 4 Faculty of Medicine and Odontology, University of Valencia, 46004 Valencia, Spain; 5 Biomaterial Engineering, Catholic University of San Antonio de Murcia (UCAM), Av. de los Jerónimos, 135, 30107 Murcia, Spain; 6 Villa Serena Foundation for Research, Via Leonardo Petruzzi 42, 65013 Città Sant’Angelo, Italy; 7 Casa di Cura Villa Serena del Dott. L. Petruzzi, Via Leonardo Petruzzi 42, 65013 Città Sant’Angelo, Italy

**Keywords:** complex electro-magnetic fields, *Candida albicans*, CFU, titanium, germ tube test

## Abstract

This study evaluates the effects of different programs of complex electromagnetic fields (C.M.F.s) on *Candida albicans*, in planktonic and sessile phase and on human gingival fibroblasts (HGF cells). In vitro cultures of *C. albicans* ATCC 10231 and HGF cells were exposed to different cycles of C.M.F.s defined as: oxidative stress, oxidative stress/antibacterial, antibacterial, antibacterial/oxidative stress. Colony forming units (CFUs), metabolic activity, cells viability (live/dead), cell morphology, filamentation analysis, and cytotoxicity assay were performed. The broth cultures, exposed to the different C.M.F.s, were grown on titanium discs for 48 h. The quantity comparisons of adhered *C. albicans* on surfaces were determined by CFUs and scanning electron microscopy. The *C. albicans* growth could be readily controlled with C.M.F.s reducing the number of cultivable planktonic cells vs. controls, independently by the treatment applied. In particular, the antibacterial program was associated with lower levels of CFUs. The quantification of the metabolic activity was significantly lower by using the oxidative stress program. Live/dead images showed that C.M.F.s significantly decreased the viability of *C. albicans*. C.M.F.s inhibited *C. albicans* virulence traits reducing hyphal morphogenesis, adhesion, and biofilm formation on titanium discs. The MTS assay showed no negative effects on the viability of HGF. Independent of the adopted protocol, C.M.F.s exert antifungal and anti-virulence action against *C. albicans*, no cytotoxicity effects on HGF and can be useful in the prevention and treatment of yeast biofilm infections.

## 1. Introduction

The antimicrobial resistance is one of the most challenging problems of recent times [1]. It has been calculated that every year 700,000 deaths across the world are directly correlated to this trouble and recent estimates expect an increase of this number [2]. This worrying global phenomenon, today involves a wide number of microorganisms including human pathogenic yeasts such as *Candida* spp. [3]. Moreover, especially for the current available antifungal drugs, their use may be affected both by their toxicity and drug interactions that, themselves, can limit the effectiveness of the therapy [1]. Consequently, new innovative strategies are required and currently many efforts in different fields like culturomics, genomics, and bioinformatics allow us to gain further knowledge to overcome this problem [2].

Novel technologies, like light-based devices, could represent additional instruments to supply the increasing of the antibiotic resistance [4,5].

In previous studies, we have shown that light emitting diodes (LEDs) at 880 nm could provide an antibacterial effect on *Enterococcus faecalis*, a very resistant pathogen that is usually the cause of primary and secondary endodontic failures, both in the planktonic and in the biofilm aggregation form [6,7,8]. In particular, this antibacterial action seemed to increase over time [7].

LED irradiation was also effective in providing a significant decrease of *Pseudomonas aeruginosa*, an opportunistic multidrug-resistant pathogen that is one of the major causes of nosocomial infections [9]. The photodynamic therapy, with the adjunctive use of aminovulevulinic acid and red led showed interesting results on Gram positive and negative bacteria [10].

Another technology that could have great potentiality for what concerns antibacterial and antifungal activity, is the electromagnetic fields emitting (EMF) device [11,12,13]. EMFs can be generated by electrical energy of different voltage and many therapeutic treatments can be produced, differing from frequency, intensity, duration of exposure, pulses, and waveform [14].

It has been shown that the exposure to moderate static EMF is able to induce cell modifications in shape, surface, and cytoskeleton; moreover, other parameters, like the time of treatment and the modality of emission should be considered in addition to the intensity of EMFs [15]. The mechanisms of action of each EMF are not yet completely understood and there is still contrast in literature, due to the multitude of approaches and protocols that can be adopted with EMFs. In particular, the emitted fields can be static or pulsed, with signals that could differ for the duration, frequency, and intensity that can be weak (<1 mT), moderate (1 mT to 1 T), strong (1–5 T), or ultra-strong (>5 T) [14,15].

Considering that, the inhibitory effects promoted by EMFs on bacteria are also dependent on the frequency and duration of exposure, the investigation of novel protocols specific for each species could permit to obtain a more efficient therapeutic action.

Among pathogen yeasts, *C. albicans* and non-*albicans Candida* (NAC) species are the most frequently isolated yeasts from biological samples [16]. The medical impact of *C. albicans* and some of the NAC species, such as *C. tropicalis*, *C. auris*, *C. parapsilosis*, or *C. dubliniensis*, depends, in part, on their ability to form biofilms, communities of adhered cells containing a mixture of yeast, pseudohyphal, and hyphal cells encased in an extracellular matrix [17]. In particular for *C. albicans*, filamentation is a key virulence factor and this is positively correlated with adhesion and biofilm formation [18].

*Candida* spp., are commensal microorganisms and, in general, do not cause any notable damage in healthy individuals; however, in certain conditions they are able to produce different diseases [19]. In particular, in oral environments, *C. albicans* and NAC species infections result in mucosal diseases (denture stomatitis), the decay of tooth enamel (cavities), and in the failure of dental implant devices [20,21,22].

*Candida albicans*, following prolonged exposures to antifungals easily develops resistance or tolerance becoming a challenge for clinicians that need new therapeutical perspectives.

Nowadays, few data are available regarding the impact of EMF on planktonic or sessile growth of pathogenic yeasts.

The objective of this study was to investigate the in vitro effects of the exposure of *C. albicans* and human gingival fibroblasts to different programs emitting pulsed EMFs characterized by a remarkable harmonic richness, different intensity, frequency, duration. And type of wave (Machines Codes), all of which account for the denomination of “complex magnetic fields, C.M.F.s”.

To do this, planktonic cultures of *C. albicans* were exposed to C.M.F.s and analyzed for viable count, metabolic activity, cell morphology, and filamentation analysis; the human gingival fibroblasts for cytotoxicity effects. Then, considering the pathogenic role of *C. albicans* in oral cavity, the microorganism ability to adhere to titanium surfaces was evaluated after expositions to C.M.F.s.

## 2. Materials and Methods

The experiments were performed with an ambient magnetic field of 50 μT.

### 2.1. Complex Magnetic Fields Source

The C.M.F. machine, Slim version (Medicina Fisica Integrata, M.F.I., Rome, Italy), is an electronic device realized for the application of complex electromagnetic fields to biological tissues. The device emits pulsed electromagnetic fields falling within 0.1 and 250 µT.

**Figure 1:**
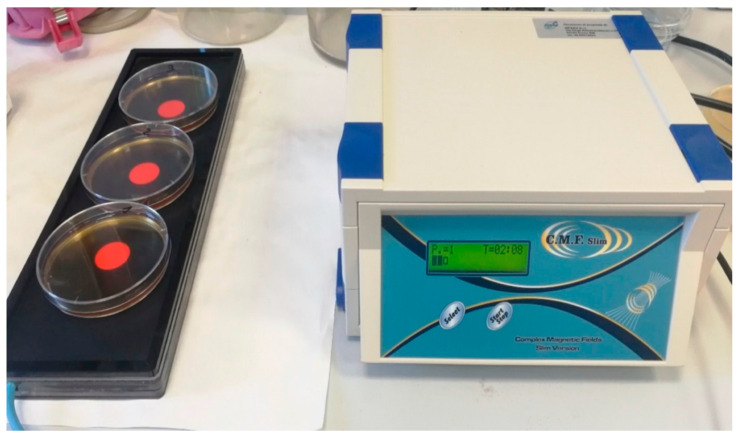
Culture samples of *Candida albicans* ATTC 10,231 during the treatment with complex magnetic fields (C.M.F.s).

Multi-frequency magnetic fields of variable intensity, frequency, duration, and wave form, were administered on culture samples of *C. albicans* ATCC 10231, by means of a series of transducers set within a mat, as shown in Figure 1. The C.M.F. generator is provided with different programming possibilities which work in relation to the configuration of the specific sector of application. As reported by the manufacturer (M.F.I.), each program is composed by a different sequence of small single steps of complex magnetic fields (3–5 min each), with frequency ranging between 6 and 70 Hz, intensity between 6 and 95 microT, and complex waveforms with multiple harmonics.

The magnets are formed by a winding of 650 turns of enameled copper wire with a 0.35 mm section. External dimensions of the coil 110 mm, internal dimensions of the coil 12 mm, thickness of the coil 8 mm. Magnetic field lines are applied at a 90 degree angle to the sample under treatment. Each step of the programs is focused on a specific fungal function to be interfered with [23].

### 2.2. Fungal Culture Conditions and Experimental Design

The reference strain *C. albicans* ATCC 10231 stored at −80 °C in cryovials containing Tripticase soy broth (TSB, OXOID, Milan, Italy) plus 10% glycerol (OXOID) was spread in Sabouraud dextrose broth (OXOID) at 37 °C. Overnight suspension was cultured 24 h at 37 °C in Sabourad-Agar [24].

The suspension of the broth culture was standardized using a spectrophotometer (Eppendorf, Milan, Italy) to obtain an optical density at OD_600_ ≃ 0.7 corresponding to 10^7^ colony forming units/mL.

Then, aliquots of 20 mL were dispensed in triplicate into Petri plates for each treatment group (Figure 1):

(A) Oxidative Stress: was subjected to one session of Oxidative Stress program with complex electromagnetic fields (C.M.F.s) for a total of 22 min of exposure.

(B) Oxidative Stress plus Antibacterial: was subjected to one session of Oxidative Stress program followed by one session of Antibacterial program of C.M.F. for a total of 44 min of exposure.

(C) Antibacterial: was subjected to one session of Antibacterial program with C.M.F. for a total of 22 min of exposure.

(D) Antibacterial plus Oxidative Stress: was subjected to one session of Antibacterial program followed by one session of Oxidative Stress program of C.M.F. for a total of 44 min of exposure.

UE: control of unexposed *C. albicans* ATCC 10231.

### 2.3. Determination of Colony-Forming Units (CFU)

For the evaluation of viable and culturable cell count, at the end of each treatment, CFU was determined by plating on Sabouraud-Agar 100 µL of serially diluted 1:10 in PBS (Becton Dickinson, Franklin Lake, NJ, USA). Then, the samples were incubated at 37 °C for 24 h and the number of colonies forming units per milliliter (CFU/mL) was determined by calculating the average count of three agar plates for dilution.

### 2.4. Viability Test

For the evaluation of cells viability, culture samples of *C. albicans* ATCC 10231 were examined with a BacLight LIVE/DEAD Viability Kit (Molecular Probes, Invitrogen detection technologies, Eugene, OR, USA). SYTO 9 stains viable cells with a green fluorescent signal, and propidium iodide stains cells with impaired membrane activity red. Then, attached fungi were washed with PBS and stained for 15 min at room temperature in the dark, as indicated by the manufacturer. The images observed at fluorescent Leica 4000 DM microscopy (Leica Microsystems, Milan, Italy) were recorded at an emission wavelength of 500 nm for SYTO 9 (green fluorescence) and of 635 nm for propidium iodide (red fluorescence). The evaluation was performed by three blinded microbiologists through the examination of at least 10 fields of view each, in a random way [25].

### 2.5. Metabolic Activity

Metabolic activity was carried out by adding chromogenic p-iodonitrotetrazolium violet (INT) (Sigma, Milan, Italy) to portions of culture samples in the microplate wells at final concentrations of 0.4 mM/L and incubated at 37 °C for 10–30 min. The spectrophotometric quantification of INT formazan concentration in each sample was performed by measuring the absorbance at 485 nm wavelength, as previously described [26].

### 2.6. Germ Tube Screening Test

In order to determine the effects of the C.M.F.s on the yeast-to-hipha transition, the suspensions of *C. albicans* ATCC 10231 from overnight cultures in Sabouraud-Agar were prepared in NYP medium (Oxoid). The test was carried out as previously described by Alves et al. [27].

The suspensions were adjusted to obtain a density of yeast cell suspensions at OD_600_ ≃ 0.7 CFU/mL and distributed into glass test tubes in a volume of 1 mL. Each dilution was exposed to different programs of C.M.F.s. After incubation at 37 °C without agitation for 3 h, the exposed and unexposed yeast cells were subjected to microscopic examination under a magnification of 100× for the presence/absence of germ tubes. A cell was considered positive for germ tube formation when the germinating tube was at least as long as the diameter of the blastospore.

The length (μm) of hyphae was measured using Leica Application Suite (LAS) software.

### 2.7. Saliva Collection

To simulate the oral cavity environment, the experiments on titanium surface were performed in presence of saliva. Human saliva was collected from healthy volunteers, who agreed to participating in the study after not drinking or eating for 2 h, as previously described [28].

Collection and use of saliva were approved by the Ethics Committee of University “G. d’Annunzio”, Chieti-Pescara, Italy (approval code SALI, N. 19 of the 10 September 2020).

Exclusion criteria included oral disease (caries or periodontitis); dental care in progress; assumption of antibiotics for three months prior to the beginning of the study. Saliva samples were mixed, clarified by centrifugation at 16,000× *g* for 1 h at 4  °C, and sterilized through low protein-binding filters (pore sizes of 0.8 μm, 0.45 µm, and 0.2 μm). The sterility of the saliva was verified by incubation in the TSB (Becton Dickinson, Franklin Lake, NJ, USA) agar plates. Saliva was considered to be sterile if no growth could be detected after 72 h of incubation at 37 °C in both aerobic and anaerobic atmospheres.

Sterile saliva was stored at −20 °C and processed within two days [28].

### 2.8. Cultivation of Candida albicans on Titanium Surface

A total of 68 IV grade titanium discs with machined surface were placed in 75% ethanol for 60 min, dried in sterilized clean bench, and then irradiated on both side with ultraviolet light for 30 min. A one-hour waiting period was observed after sterilization to allow the surfaces to equilibrate, as UV exposure has been shown to increase surface free radicals within the protective oxide layer on titanium [29].

Then, the sterilized titanium discs were placed in 96-well polystyrene microtiter plates and conditioned for 2 h by gentle shaking at room temperature with human saliva and finally washed with PBS. At this point, titanium discs were inoculated with 200 µL of *C. albicans* ATCC 10231 suspensions from the different groups: A, B, C, D and UE, and incubated at 37 °C for 48 h under aerobic conditions.

Negative controls, consisting of non-inoculated titanium discs, were also prepared.

After incubation, fungal suspensions were carefully removed and the samples were washed three times with PBS to remove non-adherent *C. albicans* ATCC 10231 cells.

For SEM analysis, a total of 14 discs were fixed with 2.5% glutaraldehyde in phosphate saline solution (PBS) and dehydrated with increasing alcohol concentrations.

The other 54 discs were placed in a sterile test tube containing 1 mL PBS and treated with ultrasonic oscillation (Euronda, Italy) for 4 min and vortexed for 2 min to remove the fungal adhering to the material surface. The effectiveness of this treatment in terms of cell aggregation and viability preservation was detected through viable staining and optical fluorescence microscopic observation. Then, selected 10-fold dilutions were plated on Sabourad-Agar plates and incubated overnight al 37 °C, followed by counting of CFU/mL. The number of adhesive yeasts on the surface of the specimen was calculated to evaluate the *C. albicans* ATCC 10231 ability to colonize the titanium surfaces.

### 2.9. Scanning Electron Microscope Observation (SEM)

Before starting with SEM observation, a Desk Sputter Coater (Phenom-World B.V., Eindhoven, The Netherlands) was used to sputter the samples with gold (150 A).

A Phenom ProX scanning electron microscope (Phenom-World B.V., The Netherlands) was used to characterize all samples at microscale and to visualize the biofilm formation on the discs.

### 2.10. Cytotoxicity Assay

Human gingival fibroblasts (HGF cells) from ATCC (Manassas, VA, USA) were cultured in DMEM low glucose (Corning, New York, NY, USA) supplemented with 10% fetal bovine serum (FBS), 1% penicillin and streptomycin at 37 °C in a humidified atmosphere of 5% CO_2_. HGF cells were seeded into 96-well/plates at a density of 6 × 10^3^ cells/well. After 24 h of culture, cells were exposed to magnetic fields as previously described in this study. At one day post treatment 10 µL/well of 2-[2-methoxy-4-nitrophenyl]-3-[4-nitrophenyl]-5-[2,4-disulphophenyl]-2H-tetrazolium, monosodium salt (MTS) assay (Promega, Madison WI, USA), was added at 37 °C for 1 h. The absorbance was measured using a microplate reader (Synergy H1 Hybrid BioTek Instruments) at 490 nm. Five replicates and three independent analysis were assessed.

The results were expressed in the form of percentage comparing each group test to the unexposed cells (UE).

### 2.11. Statistical Analysis

Data were obtained from at least three independent experiments performed in triplicate.

Results were recorded and scheduled in an Excel database (Microsoft Office 2013, Redmond, WA 98052, USA) and CFU/mL were expressed as log_10_CFU/mL. Statistical evaluation was performed by using SPSS for Windows version 21 (IBM SPSS Inc., Chicago, IL, USA). Analysis of variance (ANOVA) and LSD test were used to compare the parameters analyzed in the study. The significance threshold was set at 0.05. Data are shown as the mean value ± standard deviation (S.D.).

For cytotoxicity assay, statistical analysis was performed using GraphPad Prism 8, and to compare the two groups T test was used.

## 3. Results

**Figure 2:**
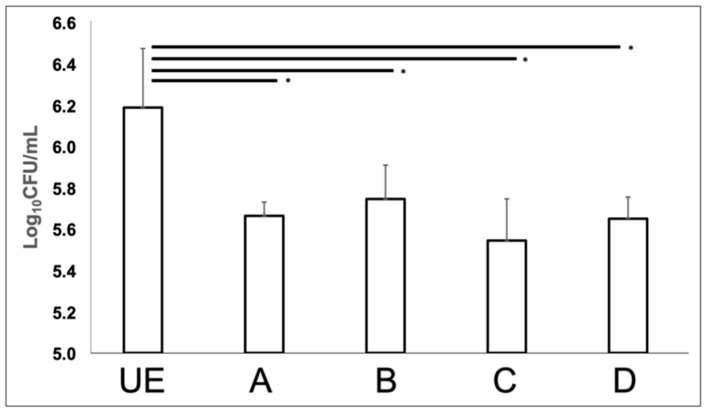
Colony forming units of *Candida albicans* ATCC 10231 after exposure to C.M.F.s. A = Oxidative Stress, B = Oxidative Stress + Antibacterial, C = Antibacterial, D = Antibacterial +Oxidative Stress, UE = control, unexposed *C. albicans* ATCC 10231. * *p*-value < 0.05.

The *C. albicans* broth cultures exposed to different program of C.M.F.s were characterized by a significant reduction of CFUs with respect to the unexposed sample (Figure 2). In particular, ANOVA analysis found a statistically significant difference (*p* = 0.001) between groups, and this result was confirmed by the LSD test that revealed a substantial reduction of *C. albicans* colony-forming units, after exposition to C.M.F.s (*p* < 0.05), independently from the program combination applied.

In detail, the lower values of CFUs were recorded by using the C/Antibacterial program (5.542 ± 0.203 log_10_ CFU/mL), followed respectively by the D/Antibacterial + Oxidative stress (5.649 ± 0.105 log_10_ CFU/mL), A/Oxidative Stress (5.662 ± 0.068 log_10_ CFU/mL), B/Oxidative stress + Antibacterial (5.744 ± 0.164 log_10_ CFU/mL) vs. unexposed sample (6.187 ± 0.285 log_10_ CFU/mL).

**Figure 3:**
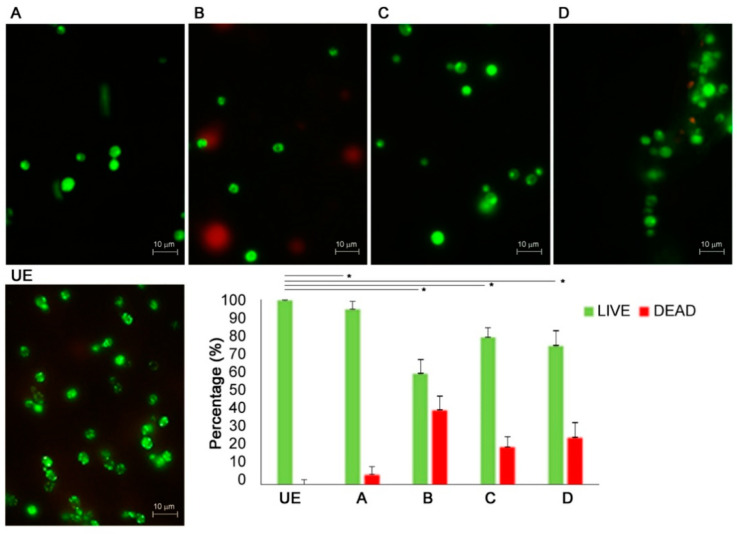
Live/dead staining of *Candida albicans* ATCC 10231 exposed to different programs of C.M.F.s. Fluorescent representative images show viable (green) and dead (red) cells after exposure to C.M.F.s with respect to the unexposed ones. Histograms show the percentages of viable and dead cells for each exposed group vs. the unexposed samples obtained with the identical methodologies in every way except for the use of C.M.F.s. The control group UE was used to evaluate the effect of each exposition (* *p*-value < 0.05). See the legend of Figure 2 for treatment groups.

Figure 3 shows the representative images of live/dead staining of *C. albicans* cells exposed to C.M.F.s different programs vs. unexposed ones.

The live/dead enumeration confirmed that the ratio between living and dead cells was significantly lower on exposed samples vs. the unexposed one. The viability of the yeasts is reduced by C.M.F.s exposition as demonstrated by mixed live and dead cells observed in exposed cultures.

**Figure 4:**
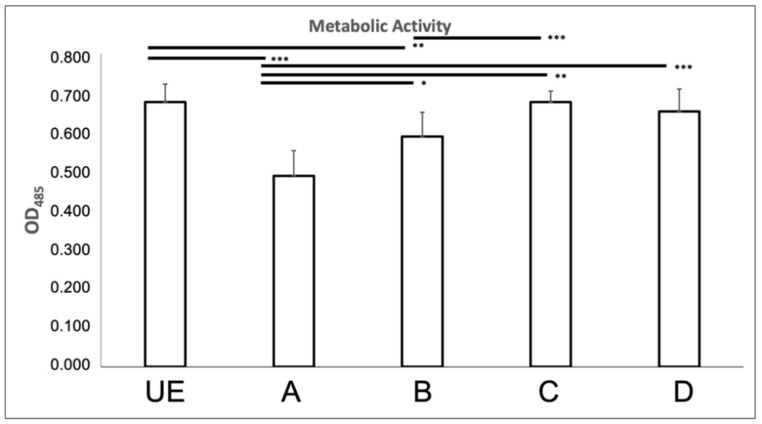
Metabolic activity of *Candida albicans* ATCC 10231 through the INT assay. See the legend of Figure 2 for treatment groups *** *p*-value < 0.01, ** *p*-value = 0.017, * *p*-value = 0.008.

The quantification of the metabolic activity of *C. albicans*, exposed to C.M.F.s through the INT assay showed a significant decrease, *p* < 0.001, after the A/Oxidative Stress program (0.488 ± 0.065), vs. C/Antibacterial program (0.676 ± 0.028) and unexposed sample (0.676 ± 0.046). The exposition to A/Oxidative stress program yielded a significant reduction of the metabolic activity also vs. the combination of Oxidative stress + Antibacterial (0.588 ± 0.062), *p* = 0.008 and Antibacterial + Oxidative stress (0.652 ± 0.057), *p* < 0.001, as shown in Figure 4.

**Figure 5:**
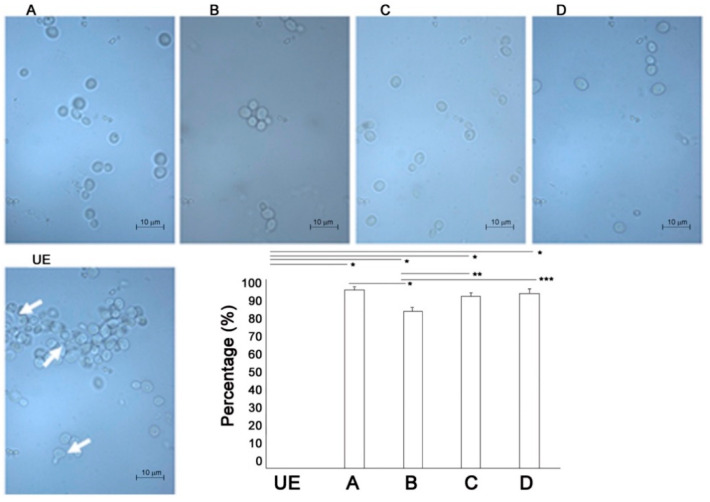
Representative images of the effect of C.M.F.s on the germ tube formation (arrows) in *Candida albicans* ATCC 10231 (microscopic examination under a magnification of 100×). Histograms show the percentage of germ tube reduction after the C.M.F.s exposure. See the legend of Figure 2 for treatment groups. * *p*-value < 0.01, ** *p*-value = 0.06, *** *p*-value = 0.002.

The effects of C.M.F.s on germ tube formation in *C. albicans* cells were analyzed by microscopic observation. As shown in Figure 5, the exposition to C.M.F.s produced a general reduction of hyphal extension arising from a yeast cell or a short hyphal extension with constriction at the point of origin, with respect to the unexposed sample, independently from the program combination applied.

The germ tube median length decreased significantly, *p* < 0.001, from 35.74 ± 14.79 μm (UE/unexposed *C. albicans*) to 10.37 ± 3.37 μm (D/Antibacterial + Oxidative stress), 4.17 ± 1.18 μm (A/Oxidative Stress), 3.75 ± 1.25 μm (C/Antibacterial program), 2.5 ± 0.50 μm (B/Oxidative stress + Antibacterial). The length of unexposed sample ranged between 63.41 μm and 19.51 μm. The minimal lengths after treatment with C.M.F.s were 0.5 μm for A/Oxidative Stress, 2 μm for B/Oxidative stress + Antibacterial, 2.5 μm for C/Antibacterial program, 5 μm for D/Antibacterial + Oxidative stress; whereas the maximal lengths were 5 μm (A), 3 μm (B), 5 μm (C), 14 μm (D).

Then, in particular, the exposition to B = Oxidative Stress + Antibacterial program have a stronger effect on the reduction on the germ tube formation in comparison to all other exposed programs of C.M.F.s.

The exposure to C.M.F.s clearly displays both the microbial growth reduction and the inhibition of germ tube formation.

**Figure 6:**
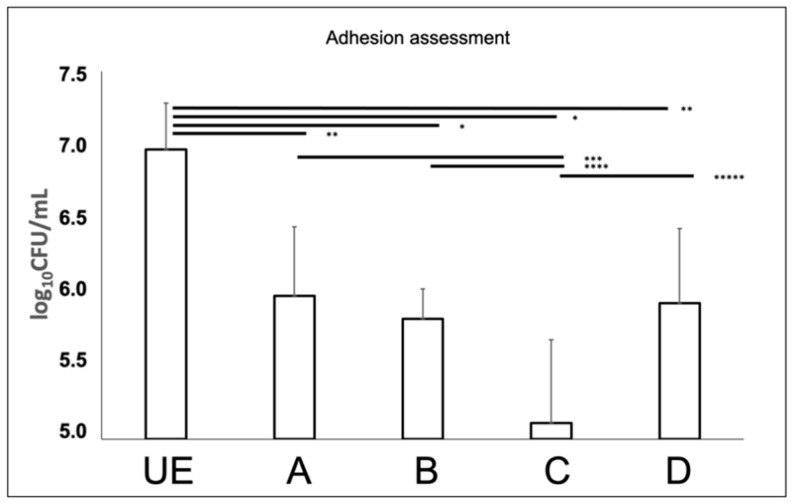
*Candida albicans* ATCC 10231 biofilm growth on saliva-conditioned titanium discs assessed by plate count agar. UE corresponds to saliva conditioned titanium disc covered by unexposed *Candida albicans* ATCC 10231. The unexposed sample was used to evaluate the effect of each exposition (* = *p*-value < 0.001; ** *p*-value = 0.001; *** *p*-value = 0.003; **** *p*-value = 0.009; ***** *p*-value = 0.005). Error bars = +/− standard deviation. See the legend of Figure 2 for treatment groups.

*Candida albicans* suspensions were exposed to different C.M.F.s programs and cultivated on titanium discs, pre-incubated with saliva, to evaluate the C.M.F.s programs effects on microbial adhesion. The count of exposed *C. albicans* adhering to surfaces showed a significant reduction of CFUs as compared to the unexposed sample (Figure 6). In particular, the lower values of CFUs were recorded by using the program C/Antibacterial (5.111 ± 0.565 log_10_ CFU/mL), followed by the B/Oxidative stress + Antibacterial (5.817 ± 0.203 log_10_ CFU/mL) D/Antibacterial + Oxidative stress (5.925 ± 0.505 log_10_ CFU/mL), A/Oxidative stress (5.975 ± 0.467 log_10_ CFU/mL), vs. unexposed sample (6.970 ± 0.316 log_10_ CFU/mL). The program C provided a significant reduction of adherent *C. albicans*, when compared to other test groups (*p* < 0.05).

**Figure 7:**
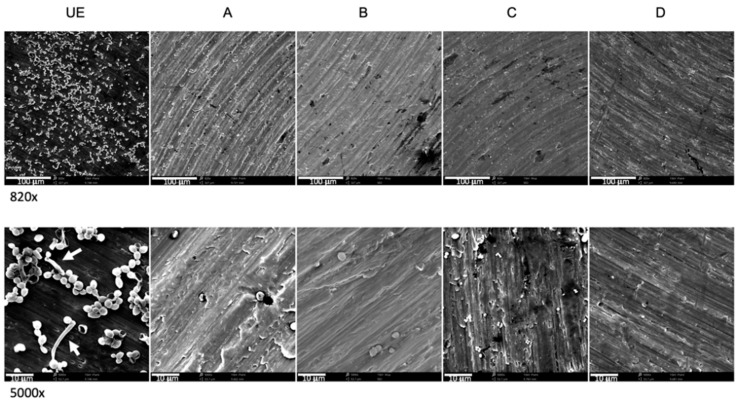
SEM images of machined titanium discs covered by adherent *Candida albicans* ATCC 10231, previously exposed to different C.M.F.s protocols, at different original magnifications, 820× (**up**) and 5000× (**down**). UE were titanium discs covered by unexposed *Candida albicans* ATCC 10231. Arrows display the germ tube presence. See the legend of Figure 2 for treatment groups.

The SEM observations, confirmed the reduction of *C. albicans* ability to adhere to titanium discs, as demonstrated by the lower quantity of yeasts observed in exposed groups (Figure 7). At 5000× magnification it can be appreciated the germ tube presence in the unexposed sample that are not present in C.M.F. group.

**Figure 8:**
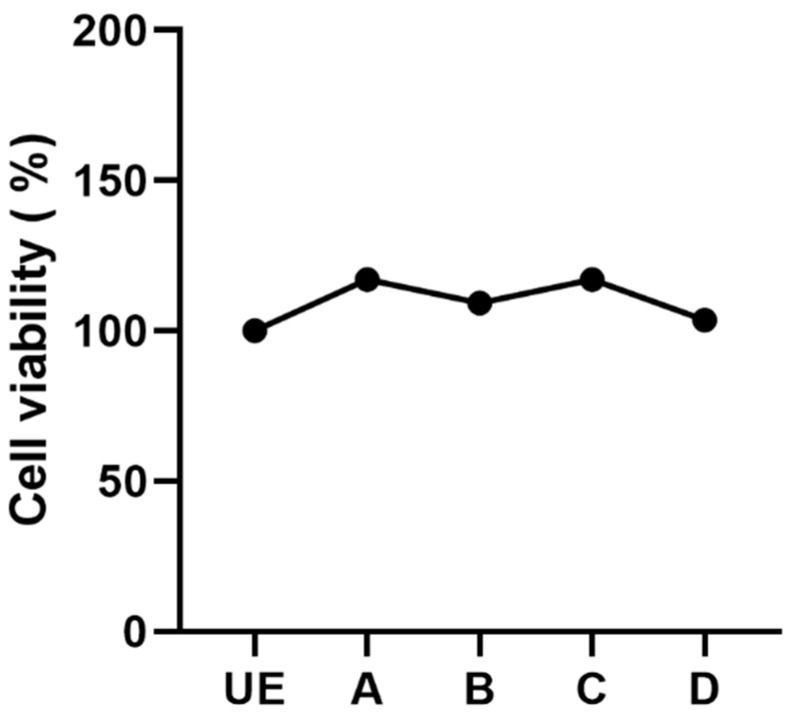
Effect of magnetic fields on HGFs viability after 24 h of C.M.F.s exposure. No cytotoxicity effects were observed within all groups. There was no significant difference between each group and unexposed cells (UE). See the legend of Figure 2 for treatment groups.

The MTS assay after 24 h of C.M.F.s exposure showed that all treatments were fully biocompatible and exerted no negative effects on the viability of HGF (Figure 8). No significant difference was found in each group with respect to the control group (UE).

## 4. Discussion

The significant increase of *Candida* spp. infections associated to the frequent failure of traditional antifungal therapy makes the study of more therapeutic alternatives mandatory [30].

*Candida albicans*, alone or in a cooperative bacterial consortium, plays a role in the development and progression of the peri-implantitis and the control of its growth in planktonic and sessile phase represents a good practice to avoid severe infections [31].

This study demonstrates the good performances of complex magnetic fields “C.M.F.s” in the significant action on *C. albicans* viability, virulence, and ability to adhere to titanium surfaces.

To simulate the oral cavity environment, the experiments were carried out on human saliva-conditioned titanium discs, since the salivary pellicle plays a central role in the establishment of the adhesion to oral surfaces and dental implants and in the subsequent biofilm formation.

The planktonic *C. albicans* culture was exposed to C.M.F.s, with different protocols that included the emission of pulsed electromagnetic fields falling between 0.1 and 250 µT interval. The capability of *C. albicans* to be cultivable and viable, detected by CFUs enumeration and by live/dead staining, significantly decreased after the exposition to C.M.F.s, independently of the adopted protocol. The differences in the amount of dead cells in the condition with the Oxidative Stress program versus the Oxidative Stress + Antibacterial could be due to the permeabilization effect which modified the cell membrane without the killing effect. This could be the reason for the difference in the live dead images considering that propidium iodide is capable to stain only dead cells. Moreover, the INT assay showed a significant reduction of metabolic activity with the Oxidative Stress program, but not after the Antibacterial one, that showed values very similar to the control.

Overall, the effect of C.M.F.s on planktonic *C. albicans* cells represents an important step toward the overcoming of the drug resistance phenomenon in *C. albicans* proposing novel intervention plans.

A recent study reported that *C. albicans* shows a 50% fluconazole and itraconazole resistance, two antifungal drugs currently used to treat candidiasis [32], supporting the need to find novel treatments as alternatives or adjuvants to the traditional ones.

In this study, the C.M.F.s exerted a reduction of *C. albicans* ability to adhere to titanium surface and also to form hyphae. A significant antiadhesive action was detected with each used protocol with a reduction of *C. albicans* adhesion on saliva conditioned titanium discs. The *C. albicans* role in the pathogenesis of peri-implantitis is of particular interest both for its ability in the development/progression of the disease and for the cooperative action with other microorganisms contributing to the formation of multispecies biofilms [17]. The reduction of the microbial colonization by opportunistic pathogens, such *C. albicans* on titanium dental implants, represents an important goal in the control of wellbeing of peri-implant tissues [21].

Moreover, the study demonstrates a significant effect of C.M.F.s on the general reduction of *C. albicans* germ tube formation, both in planktonic and sessile phase, with the strongest effect in presence of the Oxidative Stress plus Antibacterial program. In particular, the SEM analysis clearly displays a cell yeast predominant morphology in the exposed samples. This is a critical factor for fungal virulence [33], because the presence of hyphae permits *Candida* to identify and penetrate small grooves, crevices, and weak points in host tissues, to invade epithelial cells, to damage endothelial ones and, after phagocytosis, their presence can cause lysis of both macrophages and neutrophils [34]. The presence of hyphae is also a crucial element for the formation of a robust biofilm that is an essential factor for the onset of severe *Candida* infections [35].

The results obtained in this study are in accordance with Sztafrowski et al.’s, showing that treating *C. albicans* with static magnetic fields (SEF) caused a significant reduction in tail-length of the germinated cells [36].

The real EMFs mechanism of action on *C. albicans* is not fully understood. Malíková et al. described an involvement of ion channels alteration. The transfer of ions through cellular membranes is essential for the formation of the electrochemical potential of the cells and influences the signal transduction [37]. The transfer of ions through cellular membranes is essential for the formation of the electrochemical potential of the cells and influences the signal transduction. Soghomonyan et al. hypothesized that EMFs could interact with cellular membrane, causing a bacterial vibration with a consequent alteration of proteins conformation and activity [38]. Already in 2001 Binhi et al. demonstrated that bacteria under the induction of pulsed fields in the intensity range from 0 to 110 µT behaved according to the ion resonance model of A. Liboff, and that the dependence on magnetic flux density was truly extreme [39,40]. The induction of rotations promoted by the C.M.F.s signal could interfere with the possibility of creating filaments and therefore colonies and, at the same time, with the capability of duplicating, according to the model of the rotation of polar complex macromolecules [41].

In addition, after C.M.F.s treatment, *C. albicans* shows a lower ability to adhere to titanium discs. These results have a great clinical implication for dentistry, because the fungal pathogenicity is also correlated with the ability to adhere and co-aggregate [17]. In particular, it has been correlated with the development of denture stomatitis and with caries associated with braces, due to its symbiotic relationship with *Streptococcus mutans* [22,42,43,44,45]. Moreover, this fungal infection can affect mucous tissues in different parts of the body, as it happens in oropharyngeal and vulvovaginal candidiasis and in patients with a compromised immune system, like immune-deficient and cancer ones, and can be life-threatening [46].

Previous studies were performed to investigate the biological effects of magnetic fields focused on cell shape and cell viability that is an important parameter to assess cytotoxicity [47,48]. For this reason, in this study we also investigated the effects of magnetic fields exposure on HGF cells that are the most abundant cells of the connective tissue in the oral cavity. Our results showed that exposure to each kind of C.M.F.s program exerted no cytotoxic effects rather the graph of cell viability indicated a slightly proliferation after the treatments.

Overall, our data support promising important anti-virulence action associated with no cytotoxic effects for these C.M.F.s treatment, suggesting potential applications in the management of *C. albicans* infections.

The limitation of the study is related to the use of only a *C. albicans* reference strain. The main purpose of this study was to make a general evaluation of the effect of different programs of complex electromagnetic fields in a fist time against a reference strain of *C. albicans.* The interesting results obtained suggest us to carry out further studies including more *C. albicans* strains, in particular clinical azole-resistant strains, also including evaluation of the C.M.F.s contribution to the wellbeing of dental implant devices and the effect of C.M.F.s on multi species biofilms.

## 5. Conclusions

This preliminary study focused on the ability of C.M.F.s in the inhibition of planktonic growth, virulence transition into the filamentous form, and reduction of capability to adhere to titanium surface, of *Candida albicans*.

Based on these results we can affirm that C.M.F.s, at the used parameters, is a promising technology for potential applications in the treatment of *C. albicans* infections.
